# Proximity of signallers can maintain sexual signal variation under stabilizing selection

**DOI:** 10.1038/s41598-017-17327-9

**Published:** 2017-12-22

**Authors:** Michiel van Wijk, Jeremy Heath, Rik Lievers, Coby Schal, Astrid T. Groot

**Affiliations:** 1 Department of Entomology and Plant Pathology, W.M. Keck Center for Behavioral Biology, North Carolina State University, 100 Derieux Place, Raleigh, NC 27695 USA; 2 Institute for Biodiversity and Ecosystem Dynamics, University of Amsterdam, Science Park 904, 1098 XH Amsterdam, The Netherlands; 3 Max Planck Institute for Chemical Ecology, Department of Entomology, Hans Knoell strasse 8, 07745 Jena, Germany

## Abstract

How sexual communication systems can evolve under stabilizing selection is still a paradox in evolutionary biology. In moths, females emit a species-specific sex pheromone, consisting of a blend of biochemically related components, to which males are attracted. Although males appear to exert strong stabilizing selection on female pheromone, these blends seem to have evolved rapidly, as evidenced by ~120,000 moth species. Here we propose and test a “proximity model” wherein two females that vary in their relative attractiveness to males, can both benefit from calling in close proximity to each other. In a field study, we show that (1) artificially selected unattractive females can achieve mating rates comparable to attractive females if they signal in close proximity to attractive females, and (2) attractive females benefit from higher mating rates when signalling in close proximity to unattractive females. We propose that frequency-dependent behavioural and spatial interactions can sustain signal variation within populations even when these signals are under stabilizing selection.

## Introduction

A wide variety of animals solve the problem of finding sexual partners by emitting long-range sexual signals. When such signals are involved in mate choice, they are often under directional sexual selection and considerable signal variation can be expected^1–3^. However, mating signals are likely under stabilizing selection when similar signals are used in communities of closely related species. In these cases, signal variation is constrained by their pivotal role in species recognition and avoidance of hybridization^4^, as shown for example in acoustic mating signals in frogs, crickets and grasshoppers^5–9^ and chemical mating signals in moths^10–13^. It remains enigmatic, however, how signals under stabilizing selection can evolve.

Moths provide an excellent system to study this problem, as this is one of the most diverse groups of insects, comprising ~120,000 species^14^. The sex pheromones of many moth species have been identified and most consist of long-chain fatty acid derivatives including alcohols, acetates, and aldehydes^15^. Each moth species produces its own species-specific sex pheromone, consisting of a blend of biosynthetically related components at specific relative amounts^15^. For a number of moth species, male preference profiles have been found to constrain variation in female pheromone^10–13^. Even under artificial selection, inclusion of new signal variants in male preference functions can be a slow process. For example, mutant *Trichoplusia ni* females found in the laboratory produced a pheromone that contained 20-times more of a minor component (Z9-tetradecenyl acetate, Z9-14:OAc) and 30-times less of another minor component (Z5-dodecenyl acetate, Z5-12:OAc) than wild-type females^16^. In a pure colony of only this mutant phenotype, males preferred wild-type females over mutant females, and it took 49 generations of selection before males from the mutant colony showed response profiles that no longer differentiated between mutant and wild-type females^17^. Thus, even under strong positive selection for the new signal variant, the time scale of adjustment of male preference functions seems too long for the new signal to arise and persist in the population. This example illustrates how stabilizing selection appears to prevent evolutionary innovation in moth sex pheromones.

Several models have been proposed to explain evolutionary change of female sex pheromone composition in moths. The “asymmetric tracking hypothesis” assumes that females make a greater reproductive investment than males, and that male preference functions vary more than female pheromone variations to maximize the male’s ability to locate potential mating partners^18^. However, in moths it is uncertain whether males make a smaller direct investment in reproduction than females, as males can produce only one spermatophore per night, the size of each spermatophore may be 5-10% of the male’s body mass^19–21^, and the number of spermatophores in females affects female fecundity^19^, which represents an incentive for multiple matings in females. In the “wallflower” model, synchronous hatching and male polyandry causes an increased mating probability of less preferred signal variants (“wallflowers”) as the season progresses, because the prefered phenotype is depleted^22^. However, most moths emerge asynchronously and have short life spans that likely minimize the wallflower effect. Recently, the “rare male hypothesis” has been proposed, based on rare cross attraction between the Asian and the European corn borers (*Ostrinia furnacalis and O. nubilalis*), and suggests that divergent female phenotypes may attract rare males^23,24^. However, rare males can only maintain variation for female phenotypes as long as a substantial fraction of males responds to the divergent phenotype. Thus, the mechanisms that have been proposed are insufficient to explain the existence of intraspecific variation in the female pheromone signal on which selection can act.

We test a new simple model, a “proximity model”, that can explain the maintenance of variation in sexual signals based on the spatial relationship of signallers. In our model, we hypothesize that signallers in close proximity represent a better target for males than individual signallers, even if these females differ in their attractiveness to males. Two similarly attractive females calling in close proximity may benefit from increased male visits but they will also compete for the arriving male, whereas most males arriving at two differentially attractive signallers should choose the most attractive female. However, as male preference functions appear to be less restrictive in later phases of the response sequence^10,25^, some males may make “mistakes” and mate the less attractive female. We thus predict that differentially attractive females benefit more from calling in close proximity than similarly attractive females.

Several factors can drive the spatial distribution of signalling females. When local population density is high, proximity among signallers may arise spontaneously. Other factors, such as host plant fidelity or preference for exposed or elevated calling sites, may also contribute to local high densities of calling females. Auto-detection (i.e. the ability to detect same sex pheromone) is common among insect signallers that emit long-range sex pheromones, including a number of moth species^26^, and may have evolved to adjust calling behaviour, or the calling site, to the presence of conspecific signallers^27^. Also, female attraction to female pheromone has been described in trapping^28–30^ and laboratory experiments^31^, and there is one report of a lab bioassay where multiple females line up about 20 cm downwind of each other while calling^32^. Pheromonal chorusing has been reported in both Lepidoptera^27^ and Coleoptera^33^ and, as was observed in frogs^34^ and crickets^35^, the coleopteran pheromonal chorus contained non-signalling “satellite females”^36^. Since many female moths detect conspecific calling females, they may thus use this information during calling site choices.

We tested this “proximity model” in the noctuid moth *Heliothis virescens*. As in most moths, *H. virescens* females produce a long range sex pheromone to attract males^37–39^ and upon arrival, the male courts her by extending his hairpencils to release his sex pheromone^40,41^. Mating lasts 2–3 h^42^, during which the male produces a spermatophore that is ~5% of his body weight^19^. Both sexes mate multiple times, but both can only mate once per night^43^, and last male sperm precedence is about 60%^44–46^. Unfortunately, the nocturnal lifestyle of moths makes it extremely difficult to observe calling females in the field and probably accounts for the absence of field studies involving calling site choice. Through artificial selection, a sexually receptive but unattractive female phenotype (i.e., females that call and emit pheromone but do not elicit upwind flight response in males) was generated^47^. Using this selection line, we specifically tested whether (1) signallers that fail to attract males alone can achieve mating when calling in proximity of attractive signallers, and (2) attractive signallers benefit from less attractive competitors in their vicinity. We also tested whether females change their position relative to other females when calling.

## Results

In this study, we used a unique artificial selection line of *H. virescens* that produced an unattractive pheromone blend^47^. These females produce minute quantities of the two critical sex pheromone components – Z11–16:Ald and Z9–14:Ald^37,38,48^ – and instead produce large amounts of the corresponding saturated aldehydes, namely 16:Ald and 14:Ald^47^. Although these females call and emit pheromone in the field, they fail to attract males^47^, and can thus be viewed as olfactory counterparts to silent mutant crickets in acoustic communication^35^. Therefore, we refer to these females as unattractive females.

### Attractiveness of females with different 16:Ald/Z11–16:Ald sex-pheromone ratios

**Figure 1:**
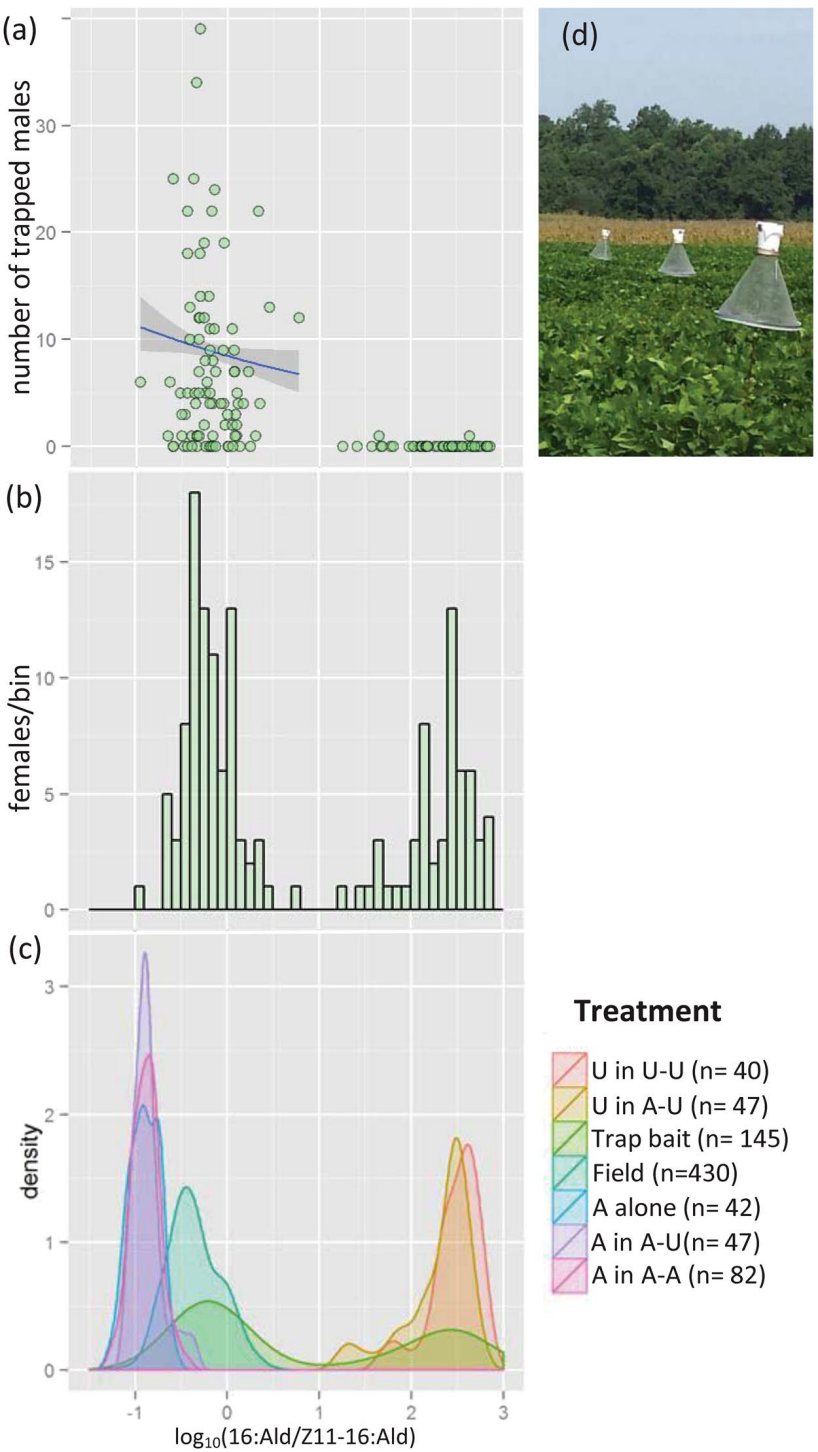
Field trapping experiments and female pheromone compositions. In panels a–c, the x-axis represents the pheromone component ratio (log_10_(16:Ald/Z11-16:Ald)) of *H. virescens* females. (**a**) Male trap catches in experiment 1. Points represent the number of males trapped at the pheromone component ratios of the females that served as lures. The regression shows that the attractiveness of wild-type females decreases with an increasing 16:Ald/Z11-16:Ald ratio. Only 2 of the 57 unattractive females (pheromone ratio > 1) trapped one male each. (**b**) Frequency distribution of the pheromone component ratio of the females that served as lures in the traps. (**c**) Geometric density plot depicting the distribution of the pheromone component ratios of females that: (1) served as lure in traps (2) were used in the mating table experiment and (3) field-collected *H. virescens* females. (**d**) Traps in the field. Legend: A: Attractive females, U: Unattractive selection line females, Trap-bait: females of the unattractive selection line (6^th^ generation), Field: *H. virescens* females collected in a soybean field.

A total of 145 females were used to attract males to traps in the field and a total of 719 males were trapped (Fig. 1a). Median trap catch was 1, and 49.6% of the traps did not catch any males. Most males (80%, 572 males) were attracted to females having a log_10_(16:Ald/Z11-16:Ald) < 0. This pheromone ratio (Fig. 1b) is within the range that is generally found in *H. virescens*, as can be seen by the distribution in the field-collected females that were collected from four different field sites in four consecutive years (Fig. 1c, see ref.^47^). The distribution of the log_10_(16:Ald/Z11-16:Ald) ratio in these females was asymmetric and a small fraction of individuals had a log_10_(16:Ald/Z11-16:Ald) > 0 (Fig. 1c). This aberrant pheromone phenotype has been found in every field population sampled^47^. Poisson regression on data points within the naturally occurring variation in the pheromone ratio of 16:Ald/Z11-16:Ald indicates that female attractiveness decreased with increasing values of the 16:Ald/Z11-16:Ald ratio (deviance = 448.11, df = 69, P < 0.04) (Fig. 1a): Females with a log_10_(16:Ald/Z11-16:Ald) < 1 were significantly more attractive to males than artificially selected females with a log_10_(16:Ald/Z11-16:Ald) > 1 (negative binomial model: deviance = 113.62, df = 143, P < 0.0001). Of the 57 traps baited with females that were later shown to have a log_10_ (16:Ald/Z11-16:Ald) > 1, only two females each caught one male (Fig. 1a).

### Can proximity to attractive females increase the mating success of unattractive females?

**Figure 2:**
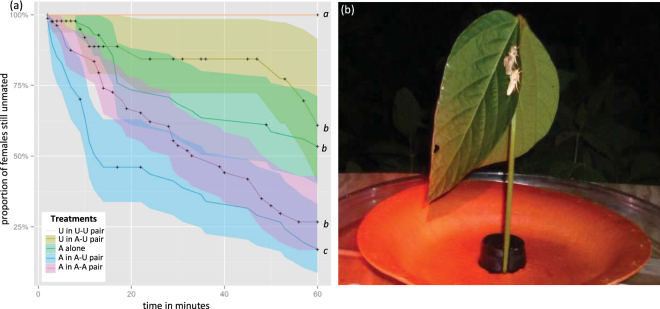
The mating table experiment. (**a**) Kaplan-Meier curves representing the mating time-course, i.e. the fraction unmated females for each treatment during the experiment. Shaded area around each curve represents 95% CI. A Cox proportional hazards model, including the time of night as a covariate, was fitted and differences between treatments were analysed using Tukey contrasts. Curves with the same letters are not significantly different according to Tukey contrasts (alpha = 0.05, see Table S1). Abbreviations as in Fig. 1, “ + ” indicates a censored data-point (unmated female that was removed, because the female that she was paired with mated, or because she had not mated within the hour). (**b**) Mating table setup consisting of a soybean branch with its terminal leaf removed and an individual, or a pair, of females with clipped wings. In the picture, the top female successfully attracted a male and is mating.

Attractive and unattractive females were placed in close proximity to one another on leaves in the field and observed until a female was mated (Fig. 2). Male availability varied with the time of night, and the Cox proportional hazards modelling confirmed that “time of night” significantly affected mating success (see Table S1 in Supporting Information). There was also a significant interaction between the treatments and “time of night” (Table S1). Similar to our trapping results (Fig. 1), we found that pairs of unattractive females failed to attract any males and none mated (Fig. 2). However, when paired with an attractive female, 17% of the unattractive females mated. Statistically, we could not discriminate between the mating time-course of unattractive females paired with an attractive female, attractive females calling alone, and pairs of attractive females (Fig. 2, Table S1).

### Can proximity to unattractive females increase the mating success of attractive females?

Attractive females in an attractive-unattractive pair mated sooner than attractive females calling alone (P < 0.05) or in pairs (P < 0.05) (Fig. 2). There was no significant difference between the mating time-course of attractive females alone and pairs of attractive females. So, for an attractive female, her time to mating was not significantly affected when calling near another attractive female, whereas calling in close proximity to an unattractive female significantly decreased her time to mating.

### At what distance is the proximity model effective?

**Figure 3:**
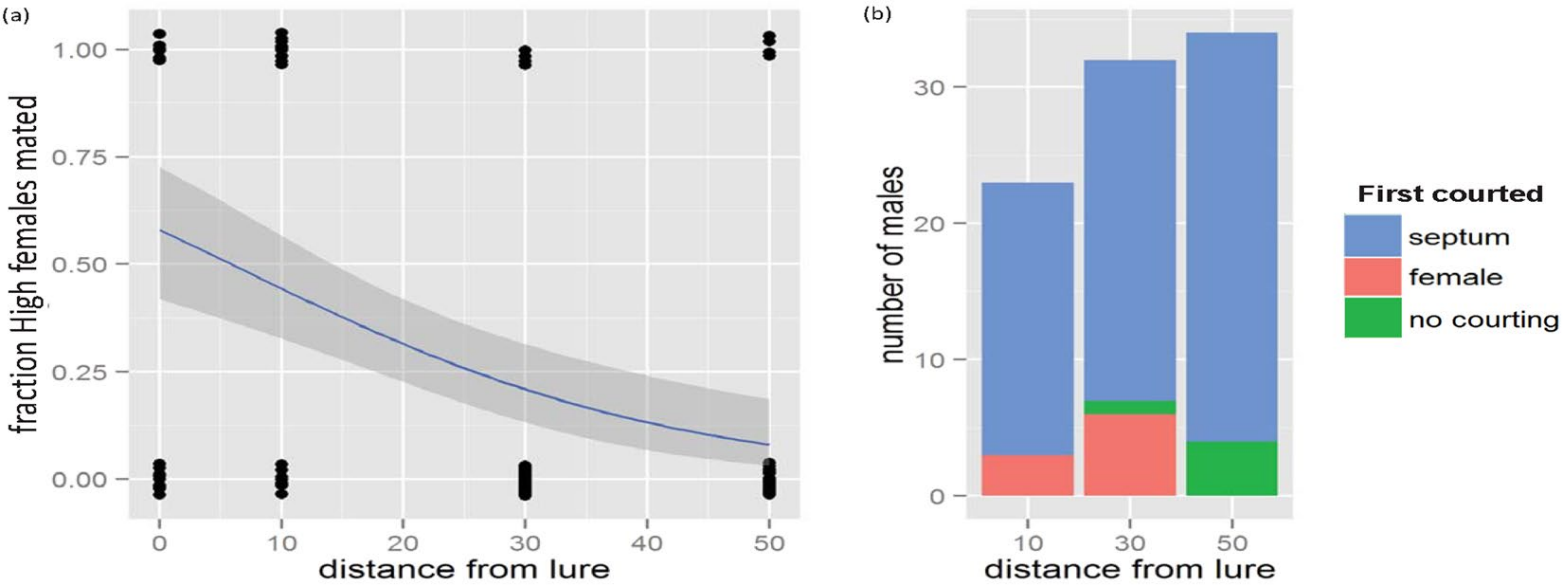
Wind tunnel experiments. (**a**) Mating success of unattractive females of the selected line decreases as a function of the distance from the attractive pheromone lure. The shaded area depicts the 95% CI of the fitted logistic regression model. Random jitter was added to the binomial response points for clarity (1 = mated, 0 = not mated). (**b**) Up to 30 cm from the lure, 13–19% of the males first courted the unattractive female.

In the wind tunnel, the mating success of unattractive females was measured as a function of their distance downwind from a lure baited with synthetic sex pheromone (Fig. 3a). The fitted logistic model had a residual deviance of 114.76 on 110 degrees of freedom. Treating this deviance as a chi-square value to test the overall fit of the model, the fitted values were not significantly different from the observed values (P = 0.36) confirming a good fit. We also recorded whether males first courted the unattractive female or the lure. This was impossible to determine at 0 cm, but at 10 cm and 30 cm downwind from the lure, respectively 13% and 18.7% of the males that flew upwind in the pheromone plume first courted the unattractive female, while at 50 cm all upwind flying males first courted the lure (Fig. 3b). One of the 38 tested males that “locked on” to a calling unattractive female made an unsuccessful mating attempt (no spermatophore was transferred) (Fig. S1a and b). There was no significant difference among the distances in the elapsed time from male release to mating (Fig. S1b, Kruskal-Wallis chi-squared = 6.09, d.f. = 3, P = 0.11).

### Does female calling affect the distance to nearest female neighbours?

**Figure 4:**
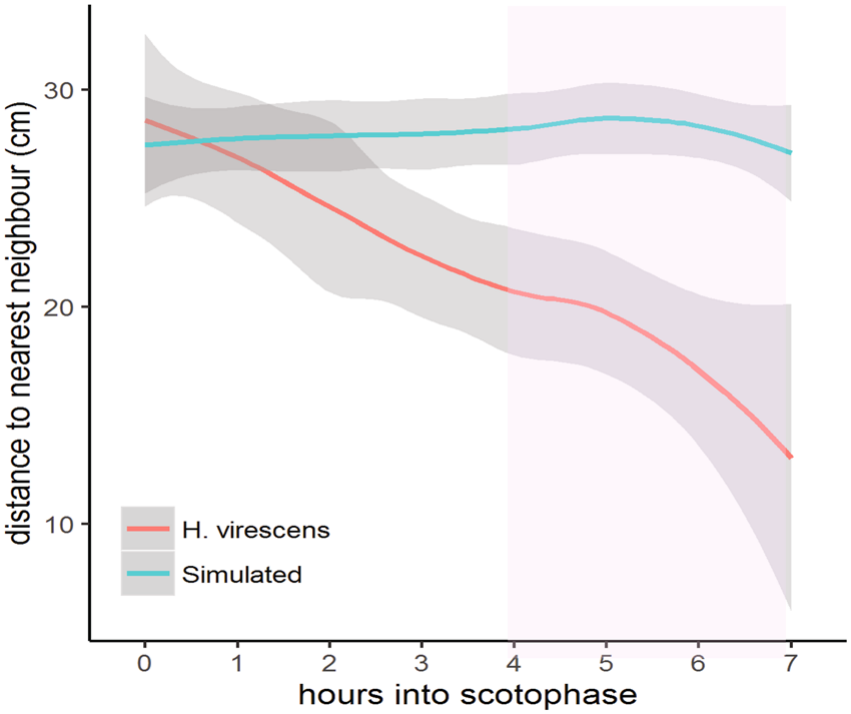
Distance to nearest neighbouring female in a large cage that contained 20 *H. virescens* females and 17, evenly distributed, flowering *Nicotiana attenuate* plants. Females are initially randomly distributed throughout the cage but as scotophase progresses and the females start calling the distance to their nearest neighbour decreases. The distribution of moths differed significantly from the simulated data only during the last four time points (Tukey contrasts) which coincides with the time that most moths are calling. Legend: *H. virescens* represents the distance to nearest neighbour of the female moths; Simulated represents the distance to the nearest neighbour in a simulated data set where a random distribution throughout the cage was assumed.

In a large cage experiment with flowering *Nicotiana attenuate* plants, the position of 20 *H. virescens* females to their nearest neighbour was compared to simulated data under the assumption that females would be randomly distributed in the cage. At the onset of the scotophase and during the following three hours, females perched on leaves and oviposited, and their positions relative to their nearest-neighbour were similar to the simulated randomly distributed females. However, 4–7 h into the scotophase most females were calling and were significantly closer to their nearest neighbour than randomly distributed simulated females (Tukey contrasts, P < 0.05) (Fig. 4).

## Discussion

In this study, we used a unique artificial selection line of female *H. virescens*. These females are sexually receptive and emit sex pheromone to attract males, but are unable to attract males from a distance. Using these females, we were able to show that male interception, possibly a “satellite” or alternative strategy, can be a viable strategy in the olfactory domain. We also found that, as females initiated calling, the distance to their nearest neighbour decreased significantly. Consistent with our proximity hypothesis, we found that signalling in close proximity is not beneficial when both females are attractive, but beneficial to differentially attractive females.

The mating frequency of the unattractive females paired with attractive females was much lower (17.0%) than of typical attractive females calling alone (45.2%) or in pairs (54.9%). However, it is important to note that our experimental design underestimates the mating potential of the unattractive females, because we always removed the pair of females as soon as one of the two females mated, which resulted in unattractive females that were paired with attractive females being removed disproportionately fast from the experiment (see Fig. 2).

Our results suggest that *H. virescens* males make navigational errors while orienting to an attractive female, which can result in mating an unattractive female calling nearby. This is in line with recent findings in the European corn borer *O. nubilalis* ^25^. *Ostrinia nubilalis* consists of two pheromone strains and males respond only to the pheromone of their own strain by starting to fly towards the odour source. However, once males have locked-on to a pheromone plume, their sensory preference widens and they continue to fly upwind even when the odour source is switched to the unattractive odour of the other strain^25^. This is the result of a generalized response to a changing perception of the pheromone ratio that results from differential adaptation to the pheromone components^25^. Adaptation may also occur in *H. virescens* males after they locked on to the attractive pheromone. It is also possible that the pheromone of the unattractive females remains unattractive, and instead, males that have locked-on to the attractive pheromone plume visually alight on the unattractive female.

Under the proximity model, it is assumed that some females may position themselves and call in close proximity to each other. Female moths perceive their own pheromone^26^, and they may use this information to position themselves relative to their nearest calling neighbour to benefit from the neighbour’s signalling. Our large cage experiment supported this hypothesis by allowing females to select resting, oviposition and calling sites on natural host plants. Similar to the findings of Otter *et al*.^32^, we found that the average distance to the nearest neighbour of calling females declined significantly (by ~50%, to <20 cm) within 4–7 hours of the onset of the scotophase, when most females were calling. How these laboratory results translate to the field remains to be investigated, but our results provide empirical support for the hypothesis that females adjust their calling position to the position of other females.

Whereas male mistakes can explain the mating success of unattractive females when in close proximity to an attractive female, mistakes cannot explain our findings that (1) attractive females mated significantly faster when paired with an unattractive female than when calling alone or when paired with an attractive female, and (2) unattractive females paired with an attractive female mated at a similar rate as attractive females alone or in pairs. Interestingly, our field and wind tunnel experiments provide two independent estimates for the magnitude of male mistakes: the mating experiments under field conditions indicate that male mistakes occurred about 17% of the time (Fig. 2b), while “first courting” in the wind tunnel experiments provided a similar range of 13–18.7% (Fig. 3b). If male mistakes were the only mechanism that allowed unattractive females to mate, one would expect an attractive female alone to mate faster than an attractive female paired with an unattractive female, because she would not experience competition from the other female and ‘lose’ 17% of the males that mate with the nearby unattractive female. Likewise, attractive females that call in close proximity to each other should experience even more competition and their time-to-mating should increase even more. These expectations contradict the experimental results and we therefore identify three non-mutually exclusive mechanisms that, in addition to mating errors, may affect mating success under the different experimental conditions: (1) Males may maximize their mating probability and preferentially orient to several females close to each other; (2) Males may reject females while taking the opportunity cost – that is, the cost of not mating this female and continue the search for a better female – into account; and (3) Competition among females may decrease sexual selection on males (See Fig. S2 for simulations of these scenarios).

### Attraction to multiple females

Males may be more attracted to a pair of females than to a female calling alone, because the odds of being outcompeted by another male are smaller or because two females represent a better close-range chemical and visual target than one female. Since we cannot discriminate between the mating time-course of attractive females alone and attractive females in pairs, we can assume that twice as many males arrive at the paired attractive females (Fig. S2c and d). If an attractive-unattractive pair also attracts twice as many males (e.g., a better visual close-range target), attractive females paired with the unattractive females thus encounter far more males than attractive females alone or attractive females in an attractive pair, as only 17% of the males make the mistake of mating with the unattractive female.

### Opportunity cost

During the experiment, we often observed several males courting the same female at the same moment, indicating that competition among males does occur in the field. Male choice may be affected by the male’s perceived encounter rate with females, which is higher when he meets a pair of females than when a male encounters a female alone. Under no-choice conditions, the perceived opportunity cost of not mating the encountered female is likely higher, because other females may be far away, which has been shown to affect sexual selection^49,50^. However, this perceived opportunity cost would increase the relative mating success of females alone compared to females in pairs, which is opposite to what we found.

### Female competition

In several systems where female mate choice has been found to be plastic, the strength of sexual selection on males positively correlated with perceived male density^51–53^. Similarly, perceived female competition might decrease the strength of sexual selection. As a male *H. virescens* arrives near a signalling female, he courts her by displaying his hairpencil pheromone glands^40,41^. Based on this behaviour, the female may accept or reject the male. Females also perceive other females^54^ and may respond to female competition by more readily accepting males or by changing their signalling behaviour^55^. If female choice contributes to the experimental outcome, an attractive female should reject about twice as many males when perceiving no competing females compared to when she is paired with another female (Fig. S2). Interestingly, this means that in systems characterized by strong female choice, intercepting females that rely on male navigational errors can increase their mating success even further by accepting all males.

Importantly, we show that simple experimental manipulation of the spatial organization of females that vary in their attractiveness to males profoundly affects their mating success. Our study is limited by the fact that we used virgin females with their wings clipped, although this was the same for all treatments and we used only females that initiated their calling behaviour. It will be very interesting to determine the generality of our findings under different conditions and in different species. Lea and Ryan^56^ recently showed that the relative preference for two signallers does not remain stable when additional options are presented: when adding a third inferior signaller to a choice test using tungara frogs, the presence or absence of this third inferior signaller reversed the relative preference for two more attractive signallers^56^. Thus, being an attractive/superior or unattractive/inferior signaller seems to be conditional on the number and quality of other signallers and their spatial organization.

Our results provide the first empirical evidence for a mechanism that can explain how divergent pheromone blends can be maintained under stabilizing selection. Male *H. virescens* likely make navigational errors after being attracted to a female from a distance, as in *O. nubilalis* ^25^, and may subsequently mate with nearby less attractive or even unattractive females, which thus relaxes the strength of stabilizing selection on the female pheromone. This effect is reinforced when male attraction increases with the number of females calling at one location, or when female sexual selection on males decreases with increasing female-female competition. In addition, our wind tunnel experiments indicate that male interception by unattractive females can be effective up to a distance of about 30 cm, although this distance may vary in the field, depending on the density of females and turbulent field conditions. As we hypothesized, both attractive and unattractive females benefitted from calling in close proximity to one another, suggesting that frequency-dependent selection may facilitate the persistence of rare, less attractive, phenotypes.

In conclusion, our results indicate that the non-uniformity of male pheromone preference functions during different phases of his search process potentially allows for surprisingly large variation in the female pheromone to persist, even though the overall male preference function appears to exert stabilizing selection on female pheromone. The persistence of female pheromone variants in the population could give males ample time to widen their response profile to new pheromone variants, as was observed in *T. ni* ^17^.

## Materials and Methods

### Insects

All experimental insects were reared in the same climate chamber, all larvae were reared individually on artificial pinto bean diet, pupae were separated by sex, and newly eclosed adults were checked daily and fed 10% sucrose water. For all experiments, virgin 2–7 day old females were used. The field trapping experiment, described below, was conducted with the 6^th^ generation of the unattractive selection line, which segregated into 50% attractive and 50% unattractive individuals, while the field experiments with the mating table and the wind tunnel experiments were conducted when the unattractive phenotype had come to fixation (17^th^ and 18^th^ generation). Control females originated from a laboratory colony (YDK) at North Carolina State University (NCSU), which contain a 16:Ald/Z11-16:Ald ratio that overlaps with field-collected females (Fig. 1c); we refer to these females as attractive females.

#### Attractiveness of females with different 16:Ald/Z11–16:Ald sex-pheromone ratios

In 2012, live females of the unattractive selection lines were used as live lures in field traps. One 2–3 day old female was enclosed in a plastic cage with gauze on both sides, which was hung in a metal screen *Heliothis* trap (Fig. 1d)^39^. Traps were at least 15 m apart and a total of 20–30 traps were placed in a tobacco field at the NCSU Central Crops Research Station Clayton, North Carolina (35°40′05.2′′N 78°30′31.3′′W). Females were deployed in the traps for 1–2 nights, after which male trap catches and the lure females were brought to the laboratory. Males were identified and counted, females were injected with *Helicoverpa zea* Pheromone Biosynthesis Activating Neuropeptide (PBAN; Phoenix Pharmaceuticals) to activate pheromone production, after which their pheromone glands were extracted and analysed, as described in ref.^57^ to determine the pheromone ratio (16:Ald/Z11–16:Ald).

#### Do unattractive females intercept males navigating to attractive females?

To determine whether unattractive females intercept males when paired with an attractive female under field conditions, newly eclosed females were briefly anaesthetized with CO_2,_ their wings were clipped, after which they were placed individually in transparent plastic containers (2 cm diam., 3 cm high) with a 10% sucrose water feeding tube, so that their calling behaviour could be observed. From 23 July to 3 August 2013, in the early evening, these virgin females were brought to a flowering soybean (*Glycine max*) plot at the same NCSU Central Crops Research Station as mentioned above. Experiments were conducted from 21.00 h–4.00 h, which included the nocturnal calling and flight activity of *H. virescens* ^58^. Experiments were conducted on horizontal mating tables (1 m^2^), onto which a freshly cut terminal branch of a soybean plant with its primary leaflet removed was placed in a rubber plug that was enclosed by a biodegradable plate (30 cm). This assembly was centered in a water filled dish (diameter 50 cm) to prevent female(s) from escaping (see Fig. 2b). A transect of eight mating tables was placed, each table 20 m apart, and perpendicular to the prevailing wind direction.

Females were transported to the field in transparent plastic containers and only entered the experiment after calling behaviour was observed (i.e., females emitting pheromone with their pheromone gland extended). Calling females were placed individually on one of the two leaves on a mating table and allowed to call for up to one hour. A mating table contained either one attractive female alone (N = 42), a pair of unattractive females (N = 40), an unattractive female paired with an attractive female (N = 47) or two attractive females (N = 82). When one female on a mating table mated, the mating female and the time of mating was recorded and the females plus the copulating male on that mating table were removed from the experiment. If available, a new pair of calling females or an individual calling female was placed on the mating table. All females were used only once. Two observers moved between occupied mating tables ensuring that each mating table was visited every 5 to 10 minutes, during which male-female interactions were noted. Each mating table was also observed continuously for 5 minutes to obtain qualitative observational data. The two observers scored formerly defined behaviours on score sheets to make sure both observers scored the same events. After the experiments, the females were brought back to the laboratory within 8 hrs and only females that contained a spermatophore were injected with PBAN followed by pheromone gland extraction for pheromone composition analysis, as described in ref.^57^.

#### At what distance from an attractive lure can unattractive females intercept attracted males?

To control for potential environmental variation, experiments to determine the distance at which unattractive females may intercept an attracted male were carried out in a wind tunnel, using a synthetic pheromone lure as the standardized attractive source. Unattractive virgin females with clipped wings were placed 0, 10, 30 or 50 cm downwind of a sex pheromone lure loaded with the typical *H. virescens* pheromone blend. Red rubber septa (Wheaton) were loaded with a total of 300 µg of a blend consisting of the following ratio (major component Z11–16:Ald set to 100%): 14:Ald (5%), Z9–14:Ald (5%), 16:Ald (10%), Z7–16:Ald (2%), Z9–16:Ald (2%), Z11–16:Ald (100%) and Z11–16:OH (1%), modified from ref.^59^. Control experiments were performed using an unattractive female without the pheromone lure. The following sample sizes were used: 0 cm (N = 42), 10 cm (N = 38), 30 cm (N = 44) and control (N = 38). The experiments were conducted 3 to 7 hours into the scotophase.

#### Are calling females randomly distributed in the environment?

It is practically impossible to assess the distribution of virgin calling females in the field because they remain immobile and cryptic under leaves and within the plant canopy, and they rapidly get mated. To determine whether the position of calling females changes relative to each other in the course of the night, 20 virgin female moths were released in a cage (200 × 90 × 90 cm) that contained 17 evenly distributed flowering *Nicotiana attenuate* plants (90 cm tall), one hour before the onset of scotophase. During the first seven hours of the scotophase, the position in cm (x,y,z) and whether the female was calling was noted for each female not in flight. The experiment was replicated 5 times. The experimental results were compared to a simulated data set wherein females were randomly distributed over the cage volume. For each real and simulated female, the distance to the nearest neighbour was determined. Experimental and simulated nearest neighbour distances were compared using nonlinear mixed-effects model, followed by Tukey contrasts.

### Statistical analyses

All statistical analyses were conducted in R version 3.0.1^60^. In the first experiment, females that were used as lures to attract males in field traps had a log_10_(16:Ald/Z11-16:Ald) distribution that was bimodal (see Fig. 1c). The attractiveness of these two phenotypes was compared by fitting negative binomial generalized linear model to the number of males caught in the different traps for females with a log_10_(16:Ald/Z11-16:Ald) < 1 (largely overlapping with the field-collected females, attractive phenotype) and females with log_10_(16:Ald/Z11-16:Ald) > 1 (unattractive phenotype). To assess how male attraction changes as a function of the naturally occurring log_10_(16:Ald/Z11-16:Ald) pheromone ratio, Poisson regression was carried out on the subset of trap catch results with a log_10_(16:Ald/Z11-16:Ald) < 1. The selected unattractive females with a log_10_(16:Ald/Z11-16:Ald) > 1 were excluded from this analysis, because their phenotype fell outside the natural variation in the log_10_(16:Ald/Z11-16:Ald) ratio. As in Groot *et al*.^47^, zero-trap-catch results were excluded from this analysis, because we cannot be sure that females in these traps actually called.

The influence of proximity to another female on the mating success of individual females was estimated by comparing the time from her introduction in the field until mating occurred. Female mating success varied over the night and therefore a Cox proportional hazards model was fitted to the treatments, with “time of night” as a covariate. Subsequently, treatments were compared to each other using Tukey contrasts. Since females from the unattractive-unattractive pairs were never mated and thus failed to meet the Cox proportional hazards model assumptions, this treatment was excluded from the model. Instead, the Kaplan-Meier curves for this treatment and the other treatments were compared, using log-rank tests. The resulting figures from this survival analysis depict traditional survival curves that we refer to as “mating time-courses”.

To analyse the distance at which males can be intercepted by unattractive females in the wind tunnel, a logistic regression model (glm) was fitted to the mating outcome as a function of the distance from the pheromone lure. To ensure that the results only reflect the probability that attracted males would mate, only males that “locked on” to the odour plume were used for this analysis. The elapsed time from male release to mating was compared among the different distances with the Kruskal-Wallis rank sum test. To assess if the “lock-on” behaviour of the males was significantly affected by the presence of the pheromone lure, a logistic model was fitted and the different distance treatments were compared to the unattractive females without lure (control), using Dunnett contrasts (Fig. S1a).

## Electronic supplementary material

Supplementary information

Dataset 1

Dataset 2

Dataset 3

Dataset 4

Dataset 5

Dataset 6

Dataset 7

Dataset 8
