# Supplementary material for: Proximity of signallers can maintain sexual signal variation under stabilizing selection
Source: Sci Rep. 2017 Dec 22;7:18101. doi: 10.1038/s41598-017-17327-9 (PMC5741759; doi:10.1038/s41598-017-17327-9)
Supplement: Supplementary file 1 — Supplementary information [file 41598_2017_17327_MOESM1_ESM.doc]

**Proximity of signallers can maintain sexual signal variation under stabilizing selection**

Michiel van Wijk, Jeremy Heath, Rik Lievers, Coby Schal, Astrid T. Groot

**Supplementary information**

**Supplementary material**

**Table S1.** Cox proportional hazards modelling of mating table experiment and the Tukey contrasts that compare the mating time-courses (Fig. 2a). The treatment where two unattractive females were paired was compared to the other treatments with a log-rank test because this treatment with zero mating did not satisfy the Cox proportional hazards criteria.

| **Cox proportional hazards model: Mating tables** | |  |  |  |
| --- | --- | --- | --- | --- |
| Treatment | N | HR | 95% CI | P |
| Attractive alone | 42 | 0.08 | 0.02 0.31 | < 0.001 |
| Attractive in Attractive – Attractive pair | 82 | 0.14 | 0.06 0.32 | < 0.001 |
| Unattractive in Attractive – Unattractive pair | 47 | 0.07 | 0.01 0.39 | 0.003 |
| Attractive in Attractive – Unattractive pair | 47 | 7.10 | 3.11 16.23 | < 0.001 |
| Time of Night (TN) | 218 | 0.97 | 0.96 0.99 | < 0.001 |
| Attractive alone: TN | 42 | 1.02 | 1.01 1.04 | 0.012 |
| Attractive in Attractive – Attractive pair: TN | 82 | 1.03 | 1.01 1.04 | 0.001 |
| Unattractive in Attractive – Unattractive pair: TN | 47 | 0.02 | 0.99 1.04 | 0.115 |
| Attractive in Attractive – Unattractive pair: TN | 47 | 0.97 | 0.96 0.99 | 0.001 |
|  |  |  |  |  |
|  |  |  |  |  |
| **Tukey contrasts: Mating tables** |  |  |  |  |
| Comparison | P |  |  |  |
| Unattractive in Attractive - Unattractive pair vs. Attractive alone | 0.999 |  |  |  |
| Unattractive in Attractive - Unattractive pair vs. Attractive in Attractive – Attractive pair | 0.812 |  |  |  |
| Unattractive in Attractive - Unattractive pair vs. Attractive in Attractive – Unattractive pair | 0.013 | * |  |  |
| Attractive in Attractive - Attractive pair vs. Attractive alone | 0.799 |  |  |  |
| Attractive in Attractive - Unattractive pair vs. Attractive alone | 0.002 | ** |  |  |
| Attractive in Attractive - Unattractive pair vs. Attractive in Attractive – Attractive pair | < 0.001 | *** |  |  |
| **Log-Rank test** |  |  |  |  |
| Unattractive in Unattractive - Unattractive pair vs. Attractive alone | < 0.001 | *** |  |  |
| Unattractive in Unattractive - Unattractive pair vs. Attractive in Attractive – Attractive pair | < 0.001 | *** |  |  |
| Unattractive in Unattractive - Unattractive pair vs. Attractive in Attractive – Attractive pair | < 0.001 | *** |  |  |
| Unattractive in Unattractive - Unattractive pair vs. Unattractive in Attractive – Unattractive pair | < 0.001 | *** |  |  |


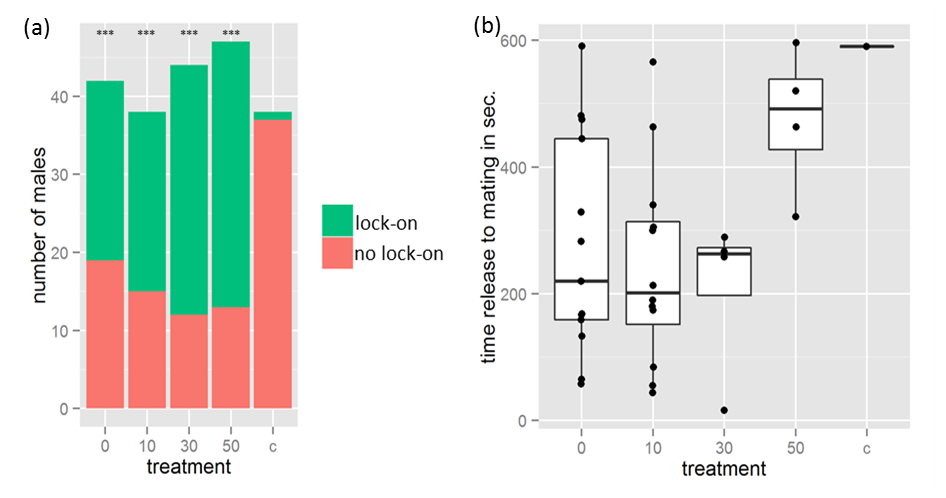


**Figure S1.** Wind tunnel experiments showing that (a) Nearly all unattractive females (c treatment) failed to elicit lock-on behaviour in the males, except for one male, which resulted in an unsuccessful mating attempt. All treatments that contained a pheromone lure differed significantly from the control (Dunnett’s contrast P < 0.001). (b) Time taken by male from release until mating. Boxes are first and third quartiles, horizontal line in the boxes represents median, vertical lines represent 95% CI of the median. Kruskal-Wallis test indicated that there was no significant difference in the time until mating between the different distances from the lure.

**Computer simulations**

A simple probabilistic simulation model was constructed in R to explore the effect of the following variables on the experimental outcome of the mating table experiments. (1) Male mistakes, i.e. the probability that a male mates a female that is in close proximity to an attractive female. Our experimental results suggest that this probability is about 17%. (2) Long-range male response, i.e. the probability that a male locks on to the pheromone plume produced by individual females or the different pairs of females in the experiments. (3) Short-range male response, i.e. the probability that a male accepts a female that calls alone or a female that is part of a pair of females close to each other. Here we assume that the perceived opportunity cost of rejecting a female that calls alone is higher than the perceived opportunity cost of rejecting a female that is part of a group of females. (4) Short-range female response, i.e. the probability that a female accepts a male, given the competition she perceives of other females in her proximity. After simulations, Kaplan-Meyer curves were generated (Fig. S2). Overall model parameters and sample size were adjusted so that simulation results and CI closely resembled the experimental outcome.


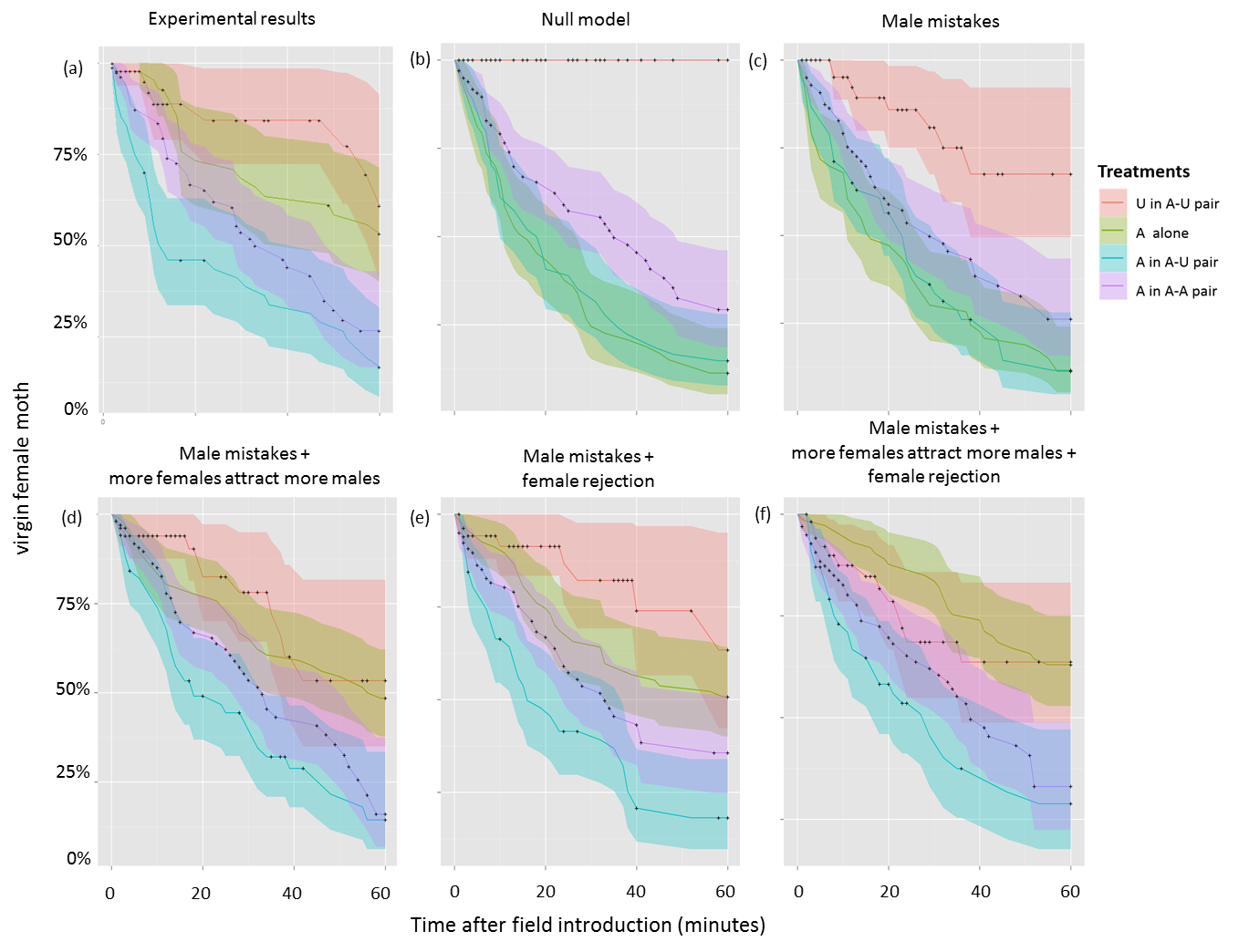


**Figure S2.** Simulation of mating table experiment. To aid in the interpretation of the experimental results, a simple simulation model was constructed. (a) The experimental results shown in Fig. 2. (b) Results of null model: males make no mistakes and are only attracted to attractive females. All females accept all males. Competition among attractive females reduces the mating time-course of the attractive females that call in close proximity. (c) Same as null model, but now 17% of the males make mistakes. (d) As null model, but 17% of the males make mistakes and a pair of females attract twice as many males as one female alone. (e) As null model, but 17% of the males make mistakes and an attractive female alone rejects twice as many males as females in pairs. (f) Combined effect of (d) and (e): As null model, but 17% of the males make mistakes and a pair of females attract twice as many males as one female alone and an attractive female alone rejects twice as many males as females in pairs. Since the CI is fairly large in the field experiments and the model was parameterized on the experimental results the scenarios (d), (e) and (f) are qualitatively similar to the results obtained in the field. Abbreviations: A: Attractive YDK females, U Unattractive females of the selection line.
